# Prostate-Specific Membrane Antigen Targeted Pet/CT Imaging in Patients with Colon, Gastric and Pancreatic Cancer

**DOI:** 10.3390/cancers14246209

**Published:** 2022-12-15

**Authors:** Floris A. Vuijk, Fleur Kleiburg, Wyanne A. Noortman, Linda Heijmen, Shirin Feshtali Shahbazi, Floris H. P. van Velden, Victor M. Baart, Shadhvi S. Bhairosingh, Bert D. Windhorst, Lukas J. A. C. Hawinkels, Petra Dibbets-Schneider, Neanke Bouwman, Stijn A. L. P. Crobach, Arantza Fariña-Sarasqueta, Andreas W. K. S. Marinelli, Daniela E. Oprea-Lager, Rutger-Jan Swijnenburg, Frits Smit, Alexander L. Vahrmeijer, Lioe-Fee de Geus-Oei, Denise E. Hilling, Marije Slingerland

**Affiliations:** 1 Department of Surgery, Leiden University Medical Center, 2333 ZA Leiden, The Netherlands; 2 Department of Radiology, Section of Nuclear Medicine, Leiden University Medical Center, 2333 ZA Leiden, The Netherlands; 3 Biomedical Photonic Imaging Group, University of Twente, 7522 NB Enschede, The Netherlands; 4 Department of Radiology, Leiden University Medical Center, 2333 ZA Leiden, The Netherlands; 5 Department of Radiology and Nuclear Medicine, Amsterdam University Medical Center, Location VUmc, 1081 HV Amsterdam, The Netherlands; 6 Department of Gastroenterology and Hepatology, Leiden University Medical Center, 2333 ZA Leiden, The Netherlands; 7 Department of Clinical Pharmacology and Toxicology, Leiden University Medical Center, 2333 ZA Leiden, The Netherlands; 8 Department of Pathology, Leiden University Medical Center, 2333 ZA Leiden, The Netherlands; 9 Department of Pathology, Amsterdam University Medical Center, Location AMC, 1081 HV Amsterdam, The Netherlands; 10 Department of Surgery, Haaglanden Medical Center, 2512 VA The Hague, The Netherlands; 11 Department of Surgery, Amsterdam UMC, Location Vrije Universiteit, 1081 HV Amsterdam, The Netherlands; 12 Department of Radiology, Alrijne Hospital, 2353 GA Leiderdorp, The Netherlands; 13 Department of Oncological and Gastrointestinal Surgery, Erasmus MC Cancer Institute, University Medical Center Rotterdam, 3015 GD Rotterdam, The Netherlands; 14 Department of Medical Oncology, Leiden University Medical Center, 2333 ZA Leiden, The Netherlands

**Keywords:** PET/CT, PSMA, colon cancer, gastric cancer, pancreatic cancer

## Abstract

**Simple Summary:**

Prostate-specific membrane antigen (PSMA)-targeted PET/CT imaging is increasingly being used for (re)staging in prostate cancer. Although PSMA suggests specificity to prostate cancer, previous preclinical studies and case reports have shown this protein to be overexpressed by multiple other tumor types. This study aims to investigate the applicability of a PSMA-targeted PET/CT tracer to detect gastrointestinal cancers, including colon, pancreatic and gastric cancer.

**Abstract:**

Current imaging modalities frequently misjudge disease stage in colorectal, gastric and pancreatic cancer. As treatment decisions are dependent on disease stage, incorrect staging has serious consequences. Previous preclinical research and case reports indicate that prostate-specific membrane antigen (PSMA)-targeted PET/CT imaging might provide a solution to some of these challenges. This prospective clinical study aims to assess the feasibility of [^18^F]DCFPyL PET/CT imaging to target and visualize primary colon, gastric and pancreatic cancer. In this prospective clinical trial, patients with colon, gastric and pancreatic cancer were included and underwent both [^18^F]DCFPyL and [^18^F]FDG PET/CT scans prior to surgical resection or (for gastric cancer) neoadjuvant therapy. Semiquantitative analysis of immunohistochemical PSMA staining was performed on the surgical resection specimens, and the results were correlated to imaging parameters. The results of this study demonstrate detection of the primary tumor by [^18^F]DCFPyL PET/CT in 7 out of 10 patients with colon, gastric and pancreatic cancer, with a mean tumor-to-blood pool ratio (TBR) of 3.3 and mean SUV_max_ of 3.6. However, due to the high surrounding uptake, visual distinction of these tumors was difficult, and the SUV_max_ and TBR on [^18^F]FDG PET/CT were significantly higher than on [^18^F]DCFPyL PET/CT. In addition, no correlation between PSMA expression in the resection specimen and SUV_max_ on [^18^F]DCFPyL PET/CT was found. In conclusion, the detection of several gastrointestinal cancers using [^18^F]DCFPyL PET/CT is feasible. However, low tumor expression and high uptake physiologically in organs/background hamper the clear distinction of the tumor. As a result, [^18^F]FDG PET/CT was superior in detecting colon, gastric and pancreatic cancers.

## 1. Introduction

Gastrointestinal cancers are among the most prevalent cancers worldwide, with colorectal cancer being the third, gastric cancer the fifth and pancreatic cancer the twelfth most common type of cancer, respectively [1]. Currently, the diagnostic workup of suspected gastrointestinal tumors includes a combination of endoscopy, computed tomography (CT), magnetic resonance imaging (MRI), [^18^F]FDG positron emission tomography–computed tomography (PET/CT), ultrasound and even diagnostic laparoscopy, depending on the tumor type. Curative treatment for all three cancers still consists of surgical resection of the primary tumor and, if indicated, chemo(radio)therapy [2].

Although these imaging modalities are frequently used in the clinic, they lack sensitivity or specificity in specific diagnostic entities, leading to over- or undertreatment. In colon cancer, for example, imaging modalities (e.g., CT) are currently insufficient in determining nodal stage. As a result, early colorectal cancers with low risk for lymph node metastases (10–15%) might currently undergo unnecessary oncologic bowel resection, while in the majority of these patients (85–90%), local treatment would suffice. In gastric cancer, the sensitivity of CT to detect distant and peritoneal metastasis is 14–65% and 22–33%, respectively [3,4,5]. Recent results from the PLASTIC trial indicated a high detection rate for the primary tumor of 79%; however, it also found the limited additional value of [^18^F]FDG PET/CT in gastric cancer staging [6]. Especially for signet cell, mucinous and poorly differentiated gastric carcinomas, [^18^F]FDG PET/CT is difficult, as they tend to be less metabolically active [7]. Even more complicating is the physiological uptake of [^18^F]FDG in the stomach wall, frequently masking the primary tumor. This results in an underestimation of the tumor stage, from which incorrect treatment choices are made. Finally, in pancreatic cancer, as much as 13% of Whipple procedures are currently being performed for benign disease [8]. Additionally, a high rate of early recurrence after resection is seen (28%) [9], indicating the presence of micro-metastases at the time of resection. Possibly, molecular imaging such as PET/CT could provide information on tumor biology.

Prostate-specific membrane antigen (PSMA)-targeted PET/CT imaging might provide a solution to some of these challenges. PSMA is a metallopeptidase that is expressed by prostate cells. Increased expression is found in prostate carcinoma, making it a well-established target for molecular imaging. PSMA-targeted PET/CT imaging has quickly evolved in the past few years and is now being adopted into the standard-of-care in the primary staging and follow-up of prostate cancer.

Recently, PSMA expression was also reported in other cancer types, including colorectal, gastric and pancreatic cancer [10,11]. PSMA expression is found on the endothelium of newly formed vasculature, which is essential for nutrient supply in all cancers. By immunohistochemical analysis, approximately 85% of colorectal cancer, 66% of gastric cancer and 84% of pancreatic cancer patients demonstrated expression of PSMA in capillaries within the tumor bed, which can be selectively targeted by [^18^F]DCFPyL [10,11]. In addition, our group demonstrated sustained PSMA expression after neoadjuvant treatment in pancreatic cancer using immunohistochemistry analysis [12]. Three case reports in patients with synchronous prostate cancer and colorectal, gastric, or pancreatic cancer suggested the feasibility of PSMA-targeted PET/CT for detection of the primary tumor and/or its metastases [10,13,14,15]. Recently, a larger study including 19 pancreatic cancer patients demonstrated positive uptake in 18 of these, and allowed for the distinction of malignant from benign pancreatic lesions, with a sensitivity and specificity of 84.2% and 90.5%, respectively [16]. Aside from being a target for molecular imaging, PSMA could also serve as a target for theranostics [17] ([^177^Lu]Lu-PSMA, [^225^Ac]Ac-PSMA).

As a first step towards the clinical use of PSMA-targeted imaging in non-prostate cancer, this feasibility study aimed to assess the feasibility of using [^18^F]DCFPyL PET/CT imaging to target and visualize primary colon, gastric and pancreatic cancer.

## 2. Materials and Methods

### 2.1. Patient Population

This is a bi-center, non-randomized prospective clinical trial. Patients admitted to the Leiden University Medical Center (Leiden, The Netherlands) and Haaglanden Medical Centrum (HMC, The Hague, The Netherlands), and diagnosed with (histologically proven) T3-4N0-2M0-1 colon, T3-4N0-2M0-1 gastric, or pancreatic cancer, were included. No sample size calculation was possible due to the exploratory nature of this study. Gastric cancer patients received neoadjuvant therapy before surgery, consisting of 4 courses of fluorouracil, leucovorin, oxaliplatin and docetaxel. The other patients (colon and pancreatic cancer) underwent surgery without prior therapy. Clinical and pathological data were obtained from medical records. No follow-up was performed. The study was conducted in concordance with the Declaration of Helsinki, and the laws and regulations of the Netherlands. The study was approved by a certified medical ethics review board (Leiden Den Haag Delft) and the local review board of the HMC. All subjects provided written informed consent prior to any study-related activities. The study was registered in the Netherlands Trial Register (NL-8919). The goal was to include 30 patients. An early stopping rule was implemented in case interim analyses after 10 patients showed lower tumor accumulation on [^18^F]DCFPyL PET/CT than on [^18^F]FDG PET/CT (significant difference in average SUV_max_ [^18^F]FDG and [^18^F]DCFPyL).

### 2.2. Data Acquisition and Image Reconstruction

As part of this trial, patients underwent both [^18^F]DCFPyL and [^18^F]FDG PET/CT prior to surgery (colon and pancreatic cancer patients) or start of neoadjuvant therapy (gastric cancer patients). There were ≥24 h between scans. [^18^F]DCFPyL was chosen due to its favorable renal clearance. All PET/CT scans were acquired on a Vereos digital PET/CT scanner (Philips Healthcare, Best, The Netherlands), except one single [^18^F]DCFPyL PET/CT scan that was acquired on a GE Discovery MI 5-Ring digital PET/CT scanner (GE, Boston, MA, USA) (the other scan from this patient was acquired on the Vereos scanner). Both PET systems are EARL-accredited. Patients underwent a low-dose CT scan (120 kV, 35 mA_eff_) for attenuation correction purposes prior to the PET scan. Patients received an average dose of 198.9 ± 38.4 MBq [^18^F]DCFPyL and were scanned after an average of 120.8 ± 5.7 min post-injection [18,19]. [^18^F]FDG was dosed using the quadratic formula with a factor of 379 MBq·min·bed^−1^·kg^−2^, resulting in an average dose of 155.8 ± 93.5 MBq [^18^F]FDG, and patients were scanned 63.4 ± 10.6 min post-injection. Before [^18^F]FDG PET/CT, patients fasted for 6 h and were prehydrated with 1 L of water. A blood glucose threshold of <11.0 mmol/L was set for patients undergoing [^18^F]FDG PET/CT. For both scans, a PET scan of the abdomen was performed in the case of colon or pancreatic cancer, and a PET scan of the abdomen to skull base was performed in the case of gastric cancer. As the detection of distant metastases or staging was not the primary aim of this study, only partial body scans were performed to minimize radiation exposure. All scans were acquired for a duration of 5 min per bed position. [^18^F]DCFPyL and [^18^F]FDG PET/CT images were reconstructed in accordance with EANM guidelines for tumor [^18^F]FDG PET imaging version 2.0 with a 4 mm³ voxel size [20].

### 2.3. Quantitative Image Analysis

PET/CT analysis was performed by two experienced, board-certified nuclear medicine physicians (L.G., L.H.) using Sectra IDS7 software (version 21.2; Sectra AB, Linköping, Sweden). The volumes of interest (VOI) were delineated using LIFEx (version 6.30; Inserm, Orsay, France) [21]. Various lesional body-weighted standardized uptake values (SUV), i.e., maximum (SUV_max_), minimum (SUV_min_), mean (SUV_mean_) and peak (SUV_peak_), as well as volumetric parameters tumor volume (TV_DCFPyL_ for [^18^F]DCFPyL or MTV for [^18^F]FDG) and total lesion uptake (TL_DCFPyL_ for [^18^F]DCFPyL or TLG for [^18^F]FDG), defined as SUV_mean_ × tumor volume), were extracted for all patients from both scans [22]. TV_DCFPyL_, TL_DCFPyL,_ MTV and TLG were determined with an isocontour set at 45% of the maximum uptake for [^18^F]DCFPyL PET/CT scans [22] and 50% of the maximum uptake for [^18^F]FDG PET/CT scans [20]. Uptake on both PET/CTs was considered positive when the SUV_max_ ≥ 2.5. Tumors were considered detectable on PET/CT imaging when a tumor-to-blood pool ratio (TBR) ≥ 2 was observed. The blood pool was delineated using a 3 × 3 pixel region of interest (ROI) in the descending aorta (the ascending aorta was not in the field of view in colon or pancreatic cancer patients) on 5 consecutive slices of the CT scan, yielding the blood pool activity used for the calculation of TBR [23]. TBR was determined by dividing the SUV_peak_ of the tumor by the SUV_peak_ of the aortic blood pool.

### 2.4. Immunohistochemistry

PSMA expression in the resection specimens (after neoadjuvant therapy in gastric cancer) was visualized using immunohistochemistry on formalin-fixed paraffin-embedded tumor tissue sections (4 µm). Endoglin was used as the gold standard for identifying activated endothelial cells [24]. After deparaffinization in xylene and rehydration, endogenous peroxidase activity was blocked with 0.3% H_2_O_2_ (20 min). Antigen retrieval was performed by boiling slides in Tris-EDTA buffer (pH 9.0) for PSMA and citrate buffer (pH 6.0) for endoglin at 95 °C (10 min), followed by overnight incubation with the primary antibodies (mouse anti-PSMA (Dako, Clone 3E6, no. N1611, 1.64 µg/mL), or goat anti-endoglin (R&D systems, BAF1097, 1.0 µg/mL)). Next, slides were incubated for 30 min at room temperature with the secondary antibodies (anti-mouse, anti-goat (Envision, Dako, Glostrup, Denmark)). Lastly, immunoreactions were visualized using 3,3′diaminobenzidine substrate buffer (Dako, Glostrup, Denmark) and counterstained using hematoxylin. Placental tissue was used as a positive control for endoglin staining, and prostate cancer tissue was used as positive control for PSMA staining. Negative controls were included in the experiments.

The evaluation of PSMA expression was performed by an experienced, board-certified gastro-intestinal pathologist (S.C.) using the semi-quantitative H-score [25,26]. This resulted in a score ranging of 0–300 and considered both staining intensity (0–3) as well as the percentage (0–100%) of target cells stained. The endoglin staining was used as the gold standard (100% staining) for neo-angiogenesis (pre-existing vasculature was excluded from the analyses by visual identification). Higher scores indicate more PSMA expression.

### 2.5. Statistical Analysis

Statistical analysis and figure editing were performed using SPSS (version 25; IBM SPSS, Inc., Chicago, IL, USA) and GraphPad Prism (version 8; GraphPad Software, Inc., San Diego, CA, USA). Due to the small sample size, all data are displayed as mean ± standard deviation. Imaging parameters of patients between [^18^F]DCFPyL and [^18^F]FDG PET/CT were compared using the independent samples *t*-test. The correlation between [^18^F]DCFPyL SUV_max_ and H-score was evaluated using a logistic regression analysis, and displayed as the r^2^ and concurrent *p*-value. A *p*-value < 0.05 was considered significant.

## 3. Results

Ten patients were included in this clinical trial in the period from August 2020 until May 2021. After the interim analysis of 10 patients, low [^18^F]DCFPyL SUV_max_ values in primary tumors compared to surrounding organs were seen in all but one patient (in contrast to high [^18^F]FDG SUV_max_ values), and the study was prematurely terminated. Six women and four men were included, who were on average 65.3 ± 11.9 years old. All patients underwent both [^18^F]DCFPyL and [^18^F]FDG PET/CT, except one (patient 5) who did not undergo the [^18^F]FDG PET/CT, as this was not part of standard-of-care diagnostics (cT2-3 gastric carcinoma). Of the 10 included patients, 4 patients were diagnosed with colon cancer, 3 with gastric cancer, and 3 with pancreatic cancer. Two patients had a well-differentiated adenocarcinoma, three were scored as well/moderate, two as moderate and three as poor. Patient characteristics are further depicted in Table 1.

### 3.1. Quantitative Analysis of PET/CT Scans

**Figure 1:**
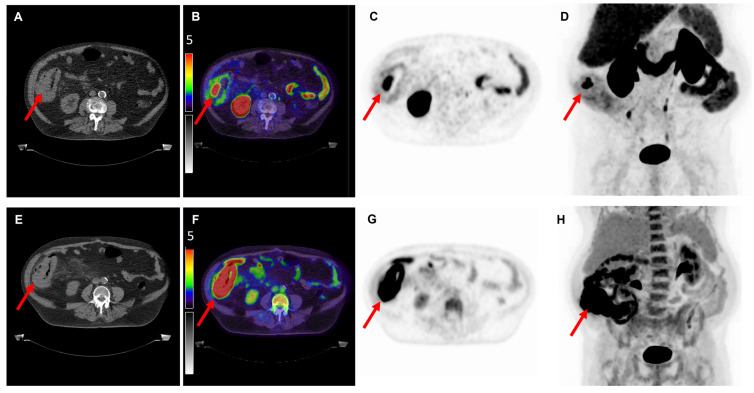
Overview of imaging modalities of a patient with pT3N0M0 colon carcinoma (patient 1). The arrows indicate (**upper row**) a lesion with intense [^18^F]DCFPyL expression with an SUV_max_ of 9.9 and (**bottom row**) a lesion with [^18^F]FDG uptake with an SUV_max_ of 45.5. From left to right: low-dose CT (**A**,**E**), fused PET/CT (**B**,**F**), PET (**C**,**G**), and the maximal intensity projection (MIP, (**D**,**H**)). Image scale SUV 0-5.

**Figure 2:**
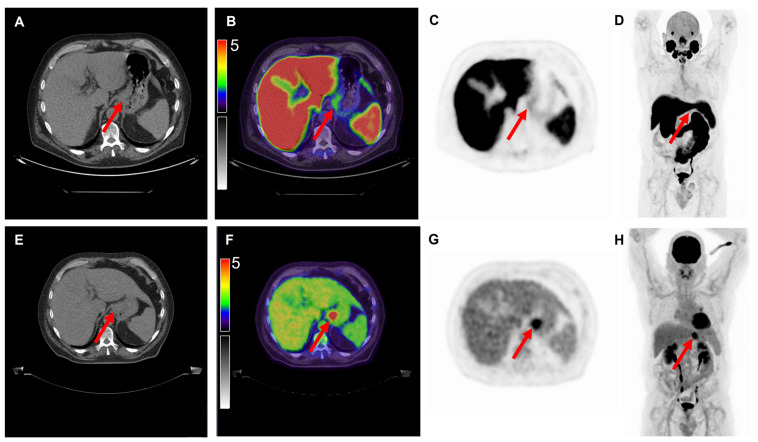
Overview of imaging modalities of a patient with cT4N1M0 tubular gastric carcinoma (patient 6). The arrows indicate (**upper row**) a lesion with light [^18^F]DCFPyL expression with an SUV_max_ of 2.5 and (**bottom row**) a lesion with [^18^F]FDG uptake with an SUV_max_ of 7.8. From left to right: low-dose CT (**A**,**E**), fused PET/CT (**B**,**F**), PET (**C**,**G**), and the maximal intensity projection (MIP, (**D**,**H)**). Image scale SUV 0-5.

**Figure 3:**
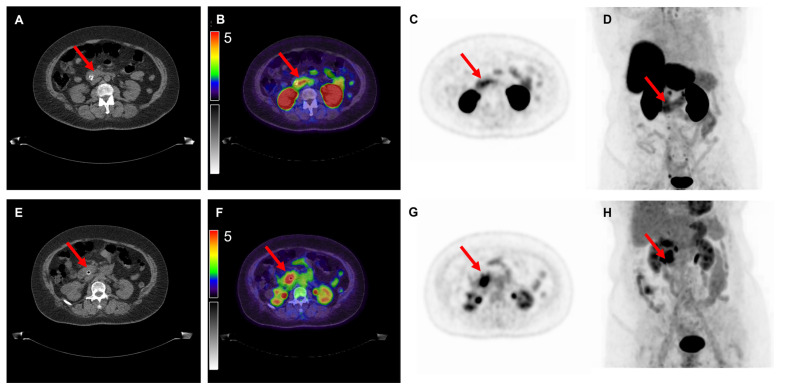
Overview of imaging modalities of a patient with pT2N2M0 pancreatic ductal adenocarcinoma (patient 10). The arrows indicate (**upper row**) a lesion with moderate to intense [^18^F]DCFPyL expression with an SUV_max_ of 5.1 and (**bottom row**) a lesion with [^18^F]FDG uptake with an SUV_max_ of 10.1. From left to right: low-dose CT (**A**,**E**), fused PET/CT (**B**,**F**), PET (**C**,**G**), and the maximal intensity projection (MIP, (**D**,**H**)). Image scale SUV 0-5.

Of the nine [^18^F]FDG PET/CT scans, 100% demonstrated positive uptake (SUV_max_ ≥ 2.5) with a mean SUV_max_ of 14.9 ± 14.5; 25.4 ± 17.0 for colon cancer, 6.1 ± 2.4 for gastric cancer and 6.8 ± 3.3 for pancreatic cancer. Of the 10 [^18^F]DCFPyL PET/CT scans, 6 (60%) demonstrated positive expression with a mean SUV_max_ of 3.6 ± 2.5; 4.2 ± 3.9 for colon cancer, 2.7 ± 0.7 for gastric cancer and 3.6 ± 1.4 for pancreatic cancer. Examples of colon, gastric and pancreatic cancer scans are displayed in Figure 1, Figure 2 and Figure 3, respectively. The primary tumor was detectable (TBR ≥2) on 6 out of 9 (67%) [^18^F]FDG PET/CT scans (3/4 colon, 1/2 gastric, 2/3 pancreatic tumors) and on 7 out of 10 (70%) [^18^F]DCFPyL PET/CT scans (3/4 colon, 1/3 gastric, 3/3 pancreatic tumors). The mean TBR on [^18^F]FDG PET/CT was 13.0 ± 8.0 for colon cancer, 2.3 ± 0.9 for gastric cancer and 3.2 ± 1.6 for pancreatic cancer.

**Figure 4:**
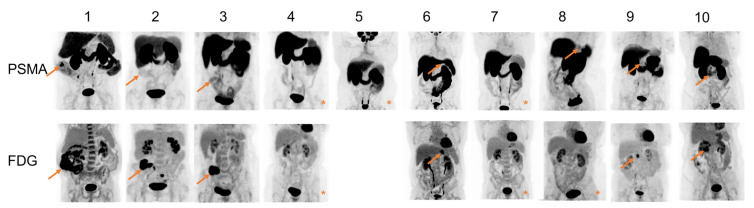
Maximum Intensity Projection (MIP) PET images of all included patients. The arrows indicate the location of the primary tumor. In the MIP PET images with an asterisk the primary tumor was not visible. [^18^F]FDG PET/CT of patient 5 was not performed as this was not the standard of care due to his cT2-3 gastric tumor. Patient numbers are identical to Table 1.

The mean TBR on [^18^F]DCFPyL was 3.3 ± 2.7 for colon cancer, 1.9 ± 0.5 for gastric cancer and 2.3 ± 0.5 for pancreatic cancer. For all patients except one (patient 1), volumetric PET/CT-derived parameters could not be extracted due to the relatively low tumor uptake of [^18^F]DCFPyL and the high uptake in surrounding tissue. The SUV_max_ and TBR on [^18^F]FDG were significantly higher compared to [^18^F]DCFPyL (*p* = 0.028 and *p* = 0.049, respectively). Although the primary metastatic sites were included in the field of view of the scans, no previously unknown lesions were found on [^18^F]DCFPyL or [^18^F]FDG PET/CT. Figure 4 shows maximal intensity projections of both [^18^F]FDG and [^18^F]DCFPyL PET/CT scans, indicating the much more intense uptake of [^18^F]FDG compared to [^18^F]DCFPyL. In one patient (patient 1), additional parameters could be extracted from both [^18^F]DCFPyL and [^18^F]FDG PET/CT. When comparing the [^18^F]DCFPyL to [^18^F]FDG PET/CT for this patient, the SUV_max_ was 9.9 versus 45.5, SUV_mean_ was 6.4 versus 28.4, SUV_min_ was 4.5 versus 22.8, SUV_peak_ was 8.4 versus 41.0, TBR was 7.3 versus 20.4, TV_DCFPyL_ was 13.6 cm^3^ versus MTV 59.4 cm^3^, and TL_DCFPyL_ was 87.6 versus TLG 1686.1, as displayed in Table 2.

### 3.2. Immunohistochemical Analysis

**Figure 5:**
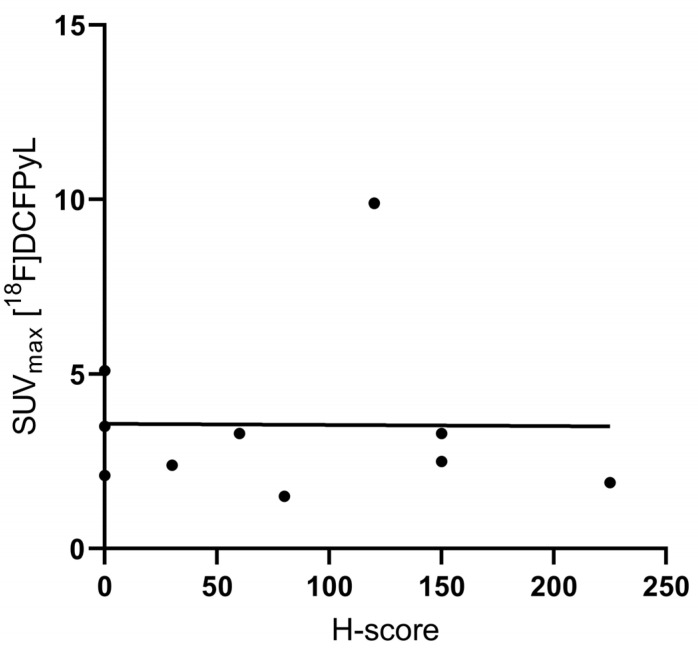
Scatterplot of [^18^F]DCFPyL SUV_max_ values with associated H scores. Abbreviations: SUV_max_, maximal standardized uptake value.

**Figure 6:**
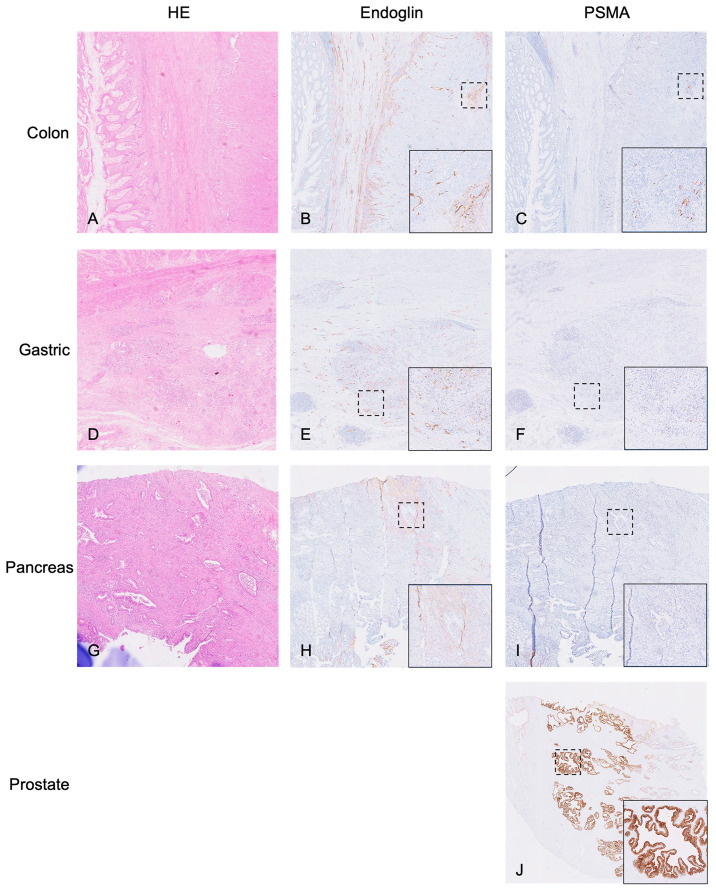
Overview of immunohistochemical stainings. This figure displays Hematoxylin and Eosin (HE), endoglin and PSMA staining of, respectively, colon ((**A**–**C**), H-score 120), gastric ((**D**–**F**), H-score 150) and pancreatic cancer ((**G**–**I**), H-score 0). As a positive control, the PSMA staining was performed on prostate cancer tissue ((**J**), H-score 300). Overview images were made at 1–2× magnification, zoom images at 10× magnification.

Immunohistochemistry resulted in a general mean H-score of 81.5 ± 77.8—121.3 ± 73.5 for colon cancer, 50.0 ± 86.6 for gastric cancer, and 60.0 ± 79.4 for pancreatic cancer. [^18^F]DCFPyL SUV_max_ was not correlated to the PSMA H-score (R^2^ 0.0001, *p* = 0.997; Figure 5). Figure 6 shows examples of immunohistochemical staining for the PSMA of the patients displayed in Figure 1, Figure 2 and Figure 3.

## 4. Discussion

Results from this study demonstrate the detection of the primary tumor by [^18^F]DCFPyL PET/CT in 7 out of 10 patients (3/4 colon, 1/3 gastric, 3/3 pancreatic cancers), with a mean TBR of 3.3 and mean SUV_max_ of 3.6. However, due to the low contrast and high level of uptake in the surrounding tissue, the visual distinction of these tumors was difficult, and the SUV_max_ and TBR on [^18^F]DCFPyL PET/CT were significantly lower compared to [^18^F]FDG PET/CT. In addition, no correlation between PSMA expression in the tumor bed in the resected specimen and SUV_max_ on [^18^F]DCFPyL PET/CT was found.

Previous literature has reported on PSMA-targeted PET tracers to detect gastrointestinal tumors. This includes incidental findings and studies with a large number of patients. In four (suspected) prostate cancer patients, colorectal cancer was unexpectedly found, with an SUV_max_ varying from 7.4 to 19.6 [13,14,15,27]. A second study, including metastatic colorectal cancer patients, found a mean SUV_max_ in three patients for the primary tumor of 7.9 ± 2.5 (using [^68^Ga]Ga-PSMA-11) [28]. This was higher when compared to our found mean SUV_max_ of 4.2 ± 3.9 in three colon cancer patients. As in our study, the SUV_max_ on [^18^F]FDG PET/CT was significantly higher than on PSMA PET/CT (23.7–43.7, *n* = 2). Unfortunately, as these patients did not undergo surgery, no correlation to PSMA expression in the resection specimen was available. Most recently, a larger study by Krishnaraju et al. including 40 patients with pancreatic lesions was conducted (21 benign (wide variety of lesions) and 19 malignant) [16]. The ^68^Ga-PSMA PET/CT was positive in 18 out of 19 pancreatic cancers, and the median SUV_max_ of malignant lesions was significantly higher compared to benign lesions (SUV_max_ 7.4 (IQR 4.5) versus 3.5 (IQR 1.6), *p* < 0.001). The sensitivity and specificity of the visual assessment of ^68^Ga-PSMA in detecting malignant pancreatic lesions were 94.7% and 90.5%, respectively. Using a quantitative SUV_max_ cut-off value of 4.8, ^68^Ga-PSMA detected malignant disease with a sensitivity of 84.2% and specificity of 90.5%. The study by Krishnaraju et al. found a considerably higher PSMA uptake in pancreatic cancers compared to our study (median SUV_max_ 7.4 versus median SUV_max_ of 3.3 in our study). Interestingly, the study by Krishnaraju et al. also performed [^18^F]FDG PET/CT in each patient; however, the median SUV_max_ values of both PET tracers were similar ([^18^F]FDG 7.6, ^68^Ga-PSMA 7.4), and the SUV_max_ values of [^18^F]FDG PET/CT were comparable to our study (mean SUV_max_ [^18^F]FDG 6.8). The difference in PSMA uptake between these studies currently remains unexplained, but could be influenced by the differences in pharmacokinetic properties and targeting characteristics (e.g., affinity, binding site) between [^18^F]DCFPyL and ^68^Ga-PSMA [29,30]. In addition, no proper pharmacokinetics studies with ^68^Ga-PSMA were performed, as have been performed for [^18^F]DCFPyL (including arterial and venous sampling).

The relatively low uptake of [^18^F]DCFPyL in this study is probably due to the low PSMA expression on the tumors. As is visualized in Figure 6, PSMA expression in the tumor bed of these cancers is significantly lower compared to prostate cancer. Although the endothelial expression of PSMA was visually intense, it was only seen in a low number of angiogenic endothelial cells. However, the IHC results for colon cancer, for example, were in line with previous literature, as all four patients expressed PSMA at varying levels. The physiological uptake of [^18^F]DCFPyL in the target organs has previously been described by Giesel et al., who found a median SUV_max_ of 2.95 in the pancreas, but did not find any notable uptake in the stomach or colon (*n* = 12) [31]. [^18^F]DCFPyL is, however, the most suitable tracer for the detection of gastrointestinal cancers due to its favorable renal clearance, as its alternative, [^18^F]PSMA-1007, shows predominant hepatobiliary excretion leading to an even higher background signal in both liver and intestines, which interferes with potentially pathological tracer accumulation, especially in these cancers [31]. The low uptake of [^18^F]DCFPyL in patients with a high H-score could indicate the tracer was not able to penetrate into the tumor core enough. In general, it might be possible that higher-grade tumors (such as included in the study by Cuda et al. [28]) express higher degrees of PSMA. In addition, it is unclear what effect neoadjuvant therapy in gastric cancer patients could have had on the immunohistochemical staining of PSMA.

Possible limitations of this study include the limited sample size, which is due to the premature termination of the trial. However, results from the included 10 patients demonstrate a clear pattern of high background and low tumor uptake, hampering clear tumor identification. As these results appear to be valid for most patients, we believe these results are representative of a larger population of the selected cancer types and thereby provide relevant information. To the best of our knowledge, this is one of the first prospective studies to include patients with gastrointestinal cancers and perform both [^18^F]DCFPyL as well as [^18^F]FDG PET/CT, and provide correlation to immunohistothe chemical expression of PSMA.

## 5. Conclusions

In conclusion, the detection of colon, gastric and pancreatic cancer using [^18^F]DCFPyL PET/CT imaging is feasible. However, low tumor uptake and high uptake in other organs hamper the clear distinction of tumor mass. In this study, [^18^F]FDG PET/CT was found to be superior in detecting colon, gastric and pancreatic cancers. These results do not encourage further investigation into the application of [^18^F]DCFPyL PET/CT imaging in these cancers. However, this may be different for other PSMA-targeted tracers.

## Figures and Tables

**Table 1:** Overview of patient characteristics.

No Figure	Age	Tumor Location	Tumor Differentiation	cTNM Stage *	pTNM Stage	Max Diameter (mm) **	SUV_max_ [^18^F]DCFPyL	SUV_max_ [^18^F]FDG	TBR [^18^F]DCFPyL	TBR [^18^F]FDG	H-Score
1	72	Colon adenocarcinoma	Well/moderate	cT3/4N1M0	pT3N0M0	180	9.9	45.5	7.3	20.4	120
2	68	Colon adenocarcinoma	Well/moderate	cT4N2M0	pT4N0M0	80	1.9	29.1	2.3	15.6	225
3	73	Colon adenocarcinoma	Poor	cT4N0M0	pT4N0M0	50	3.3	22.5	2.4	14.1	60
4	58	Colon adenocarcinoma	Well/moderate	cTxN0M0	pT4N0M0	15	1.5	4.5	1.2	1.7	80
5	38	Signet ring cell gastric carcinoma	Poor	cT2-3N0M0	ypT3N0M0	42	3.5	n.a.	1.9	n.a.	0
6	71	Tubular gastric adenocarcinoma	Moderate	cT4N1M0	ypT3N1M0	25	2.5	7.8	2.3	2.9	150
7	50	Tubular gastric adenocarcinoma	Poor	cT3N0M0	ypT4N1M0	45	2.1	4.4	1.4	1.6	0
8	70	PDAC	Well	cTxN0M0	pT2N1M0	22	3.3	3.6	2.0	1.3	150
9	76	PDAC	Moderate	cTxN0M0	pT2N1M0	28	2.4	6.8	2.0	4.3	30
10	63	PDAC	Well	cTxN2M0	pT2N2M0	35	5.1	10.1	2.8	3.9	0

Abbreviations: TNM stage, tumor, nodal and metastatic status; SUV, standardized uptake value; n.a., not available; H-score, immunohistochemical staining score; PDAC, pancreatic ductal adenocarcinoma. * Pathological TNM stage for colon and pancreatic cancer patients, initial clinical TNM stage for gastric cancer patients (as neoadjuvant therapy was given after [^18^F]DCFPyL PET/CT). ** Diameter measured at pathological examination.

**Table 2:** Overview of extended imaging parameters of patient 1.

	[^18^F]DCFPyL	[^18^F]FDG
SUV_max_	9.9	45.5
SUV_mean_	6.4	28.4
SUV_min_	4.5	22.8
SUV_peak_	8.4	41.0
TBR	7.3	20.4
TV_DCFPyL_/MTV (cm^3^)	13.6	59.4
TL_DCFPyL_/TLG	87.6	1686.1

Abbreviations: SUV, standardized uptake value; TBR, tumor to blood pool ratio; TV_DCFPyL_, tumor volume on [^18^F]DCFPyL PET/CT; MTV, metabolic tumor volume; TL_DCFPyL_, total lesion uptake on [^18^F]DCFPyL PET/CT; TLG, total lesion glycolysis.

## Data Availability

The datasets generated during and/or analyzed during the current study are available from the corresponding author on reasonable request.
